# European Multicenter Study on Degradable Starch Microsphere TACE: The Digestible Way to Conquer HCC in Patients with High Tumor Burden

**DOI:** 10.3390/cancers13205122

**Published:** 2021-10-13

**Authors:** Johannes M. Ludwig, Roberto Iezzi, Jens M. Theysohn, Thomas Albrecht, Alessandro Posa, Alexander Gross

**Affiliations:** 1 Institute of Diagnostic and Interventional Radiology and Neuroradiology, Faculty of Medicine, University Duisburg-Essen, Hufeland Str. 55, 45147 Essen, Germany; johannes-maximilian.ludwig@uk-essen.de; 2 Department of Bioimaging and Radiological Sciences, Institute of Radiology, Fondazione Policlinico Universitario Agostino Gemelli IRCCS, Università Cattolica del Sacro Cuore, 00168 Rome, Italy; roberto.iezzi.md@gmail.com (R.I.); alessandro.posa@gmail.com (A.P.); 3 Department of Radiology and Interventional Therapy, Vivantes-Klinikum Neukölln, Rudower Str. 48, 12351 Berlin, Germany; Thomas.Albrecht@vivantes.de (T.A.); Alexander.Gross@vivantes.de (A.G.)

**Keywords:** carcinoma, hepatocellular, chemoembolization, therapeutic, degradable starch microspheres (DSMs) TACE

## Abstract

**Simple Summary:**

In this European multicenter study, we investigated the treatment efficacy and safety of degradable starch microsphere transarterial chemoembolization (DSM-TACE) for HCC treatment in 121 patients in whom other standard therapies failed or patients were not eligible. Patients survived a median of 15.5 months with a median time to tumor progression of 9.5 months and a disease control rate of 83.2%. The patients who survived longer had HCC lesions ≤10 cm, the involvement of one liver lobe only, lower Child–Pugh class and Barcelona Clinic Liver Cancer (BCLC) tumor stage, absence of vascular invasion, and the absence of extrahepatic metastases. Of these factors, a lesion of ≤10 cm and unilobar disease were identified as independent survival factors. Safety analysis revealed low rates of adverse events and maintained liver function after several treatments regardless of the treated liver volume. Thus, DSM-TACE is a veritable treatment alternative for unresectable HCC, where other treatments fail or cannot be offered due to contraindications.

**Abstract:**

To evaluate the safety and efficacy of transarterial chemoembolization with degradable starch microspheres (DSM-TACE) for the treatment of hepatocellular carcinoma (HCC) with a high tumor burden ineligible for or failing other palliative therapies, 121 patients from three European centers were included. Kaplan–Meier analysis was used for median overall survival (OS) and time to progression (TTP, mRECIST criteria) in months with a 95% confidence interval (95% CI). Uni- (UVA) and multivariate (MVA) analyses were performed using the Cox Proportional Hazard Model. The median OS of the study cohort was 15.5 (13.3–18.7) months. The UVA identified HCC lesions ≤10 cm, unilobar involvement, lower Child–Pugh class and Barcelona Clinic Liver Cancer (BCLC) stage, absence of vascular invasion, and extrahepatic metastases as factors for prolonged survival. MVA confirmed lesions of ≤10 cm and unilobar disease as independent OS factors. Median TTP was 9.5 (7.6–10.3) months. The best response was achieved after a median of 3 (range: 1–6) treatments with CR/PR/SD/PD in 13.5%/44.5%/25.2%/16.8%, respectively. DSM-TACE was well tolerated with no major clinical adverse events and only limited major laboratory events. Preserved liver function was observed after repetitive DSM-TACE treatments. Repetitive DSM-TACE is a safe, well-tolerated and effective treatment option for HCC patients with high tumor burden ineligible or failing other palliative therapies.

## 1. Introduction

Hepatocellular carcinoma (HCC) is the world’s fourth leading cause of cancer-related death, with increasing incidence rates and cancer-specific mortality in many countries [1,2]. Unfortunately, most patients are diagnosed at stages where ablation, resection and transplantation are no longer possible curative treatment options. For these patients, catheter-based therapies are an optional treatment method with transarterial chemoembolization (TACE) recommended as first-line therapy by the European Association for the Study of the Liver guidelines for intermediate-stage HCC patients [3]. Several studies even propagate the benefit of TACE for selected patients with early and advanced stages, further expanding the treatment indications [4].

To date, TACE with Lipiodol (conventional TACE, cTACE) or drug-eluting beads (DEBs) as embolic agents is the most commonly used option [3,4]. Despite being available for decades, degradable starch microspheres (DSMs) have only recently emerged as a viable embolic agent alternative. The most relevant difference is the well-defined and transient vessel occlusion with a half-life time of approximately 40 min for particles with 50 μm in diameter compared to the prolonged washout of Lipiodol (5–12 weeks) and the permanent vessel occlusion of DEBs [3,4,5,6]. Temporary occlusion bears several benefits, including shorter ischemia time for reduced post embolization syndrome and the ability to reperform treatment, as vessels will be patented for further transarterial treatments [7,8,9,10,11]. Liver parenchyma embolization rarely causes substantial harm in conserving healthy liver tissue [12]. Thus, as unselective embolization can be performed with high tolerability and safety rates, DSM-TACE represents a veritable option for the bilobar extensive disease or when a selective treatment cannot be performed. The purpose of this European multicenter study was to evaluate the treatment effectiveness and liver tolerability of transarterial chemoembolization with degradable starch microspheres (DSMs).

## 2. Materials and Methods

### 2.1. Study Design and Patient Population

In this retrospective European multicenter study, 121 patients with HCC from three centers were included: Vivantes Hospital Neukölln in Berlin, Germany (*n* = 37); A. Gemelli University Hospital in Rome, Italy (*n* = 56); and the University Hospital in Essen, Germany (*n* = 28). All patients have been reported previously aside from 16 new patients treated at the A. Gemelli University Hospital in Rome, Italy [8,9,10]. Patients received the first DSM-TACE treatment between September 2009 and August 2018. Approval from the ethics committee was granted, and written informed consent was waived by each Institutional Review Board. All treatment decisions were based on a multi-disciplinary consensus obtained during tumor board meetings attended by all specialties involved in the HCC patients’ management.

To be treated with DSM-TACE, patients had to have unresectable HCC with more specific inclusion and exclusion criteria for each institution. Berlin: ineligible for super-selective TACE (BCLC B) and patients with BCLC C and D if a potential clinical benefit was assumed. Rome: dismissing (tumor progression, adverse events) or ineligible for sorafenib, BCLC B refractory to TACE or BCLC C, Child–Pugh A or B, tumor burden <70%, limited extrahepatic portal/mesenteric lymph node metastases without other extrahepatic metastases, Eastern Cooperative Oncology Group (ECOG) 0–1. Essen: Not suitable for ablation, transplantation, conventional TACE (lesion count > 3, lesion size > 7 cm, decompensated cirrhosis, progression under TACE, lack of hypervascularization under fluoroscopy) or radioembolization (total bilirubin levels >2 mg/dL, high and uncorrectable hepatopulmonary shunting, reflux into arteries of the gastroduodenal region), systemic therapy with kinase inhibitors and ECOG status 0–2 and bilirubin levels up to 3 mg/dL. Further details on each institution’s inclusion and exclusion criteria can be found in the original publications [8,9,10].

The Liver Cancer Study Group of Japan Classification for the portal vein tumor thrombus (PVTT) was used, and data were stratified according to peripheral to first-order branches PVTT (vp1–3) and main portal vein trunk PVTT (vp4) [13]. Hepatic vein tumor thrombus (HVTT) was also categorized by the Japanese staging system in three categories based on the extent: peripheral (vv1); major hepatic vein (vv2); or inferior vena cava (vv3) [14].

The patient population consisted of 98 male (81%) and 23 female (19%) patients with a median age of 72 years (range: 45–88 years). HCC was diagnosed using the European Association for the Study of the Liver (EASL) imaging criteria (*n* = 90) and histopathology (*n* = 31). The majority of patients had been reported previously in single-center studies with additionally obtained and updated data collected for this study [8,9,10].

### 2.2. Treatment and Therapeutic Concept

The DSM-TACE procedure was performed using EmboCept^®^ S particles (PharmaCept, Berlin, Germany) in an angiography suite, as previously described for each participating institution [8,9,10]. Treatments were performed on a “planned” and not on a “demand” basis. DSM-TACE was performed at intervals of 2–6 weeks (Rome: 2-week intervals were used for consecutive unilobar treatment in bilobar disease; Berlin: 4 weeks; Essen: 4–6 weeks). Achieving substasis or stasis, depending on the institutional protocol, was considered the treatment endpoint. When flow (sub)stasis could not be achieved with planned dosage, additional embolization was performed with Lipiodol (Lipiodol Ultra-Fluid, Guerbet, Villepinte, France) or EmboCept^®^ S particles.

### 2.3. Assessment of Hepatic Tumor Response and Survival

Data on response analysis was based on multiphasic CT and MR imaging and was available for 119 (98.3%) patients, as two died before follow-up imaging. Response assessment was performed according to the modified Response Evaluation Criteria in Solid Tumors (mRECIST) [15]. TTP was calculated from the date of the first DSM-TACE to the date when disease progression was observed. When no progression was observed and no further follow-up imaging was performed, patients were censored using the last imaging date or date of liver transplantation. Overall survival (OS) and time to progression (TTP) were calculated from the date of first treatment until the death of any cause occurred, or patients were censored using the date when they were last seen or at the date of liver transplantation.

### 2.4. Safety Analysis

Recorded clinical adverse events were obtained from patient records. Laboratory values pre and post each treatment session were used to calculate laboratory adverse events according to the Common Terminology Criteria for Adverse Events (CTCAE) v5.0 criteria. Clinical adverse events were recorded according to the Cardiovascular and Interventional Radiological Society of Europe (CIRSE) Classification System for complications [16]. As the medication of patients affecting the prothrombin time was unknown, the analysis was performed under the assumption that no affecting anticoagulant, such as warfarin, was given. For evaluating laboratory liver values over time, obtained values before each treatment were used. Data for this analysis were available for sixty-five patients.

### 2.5. Statistics

Kaplan–Meier analysis was performed to determine the median OS and TTP in months with a 95% confidence interval (CI). For uni- (UVA) and multivariate (MVA) analysis, the Cox Proportional Hazard Model analysis was applied to calculate the hazard ratios (HR), including the 95% CI. The Pearson method was used for correlation and contingency analyses. The statistical evaluation of pretreatment laboratory values over time was performed using the mixed-effect models with the pairing of repeated measurements with Greenhouse–Geisser correction. Statistical analysis was performed using GraphPad Prism (v.8.4.2 for Mac, GraphPad Software, San Diego, CA, USA) for the mixed-effect modeling and JMP 15.0 (SAS Institute Inc., Cary, NC, USA) for all other analyses. *p*-values < 0.05 were considered statistically significant.

## 3. Results

### 3.1. Demographics

One hundred and twenty-one patients were included with a median age of 77 years, 81% males, and 97% Caucasian. The majority of HCC lesions were diagnosed based on imaging criteria (*n* = 69), followed by histology (*n* = 31) and imaging + Alpha-fetoprotein (AFP) (*n* = 21). The overall tumor burden of the population was high, with bilobar disease in 63.6%, >3 HCC nodules in 61.2%, and vascular invasion in 26.4%. Among the patients, 82.6% (*n* = 100) had at least one among the features mentioned above. Please see Table 1 for patient baseline characteristics and Table 2 for baseline laboratory values.

### 3.2. Treatment Characteristics

Five-hundred and fifty-eight (558) treatments were performed with a median of four (range: 2–12) treatments per patient. Treatment was most commonly performed via lobar (56.7%), followed by bilobar (28.1%) and selective (15.1%) embolization approaches. A median of 450 mg (range: 60–1632 mg) of EmboCept^®^ S particles were mixed with doxorubicin in 66.6% of cases (median: 50 mg), followed by epirubicin (32%; median: 50 mg) or mitomycin c (1.3%; median: 5 mg). It may be noted that three patients received treatments with doxorubicin combined with mitomycin c and doxorubicin alone at different sessions. All other patients were treated with one drug only. In 91 treatment sessions (16.3%), Lipiodol with a median of 4 mL (range: 0.5–10 mL) was administered at the end of the procedure to achieve a (sub)stasis of arterial blood flow.

### 3.3. Survival Analysis

**Figure 1:**
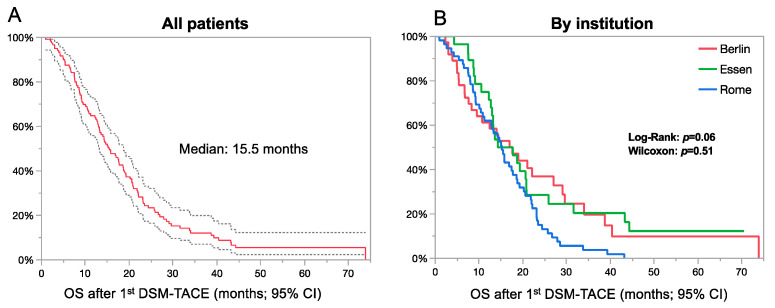
Overall survival (OS) following first DSM-TACE. OS of all patients (**A**) and stratified by institution (**B**) following first DSM-TACE. There were no statistically significant differences between institutions.

**Figure 2:**
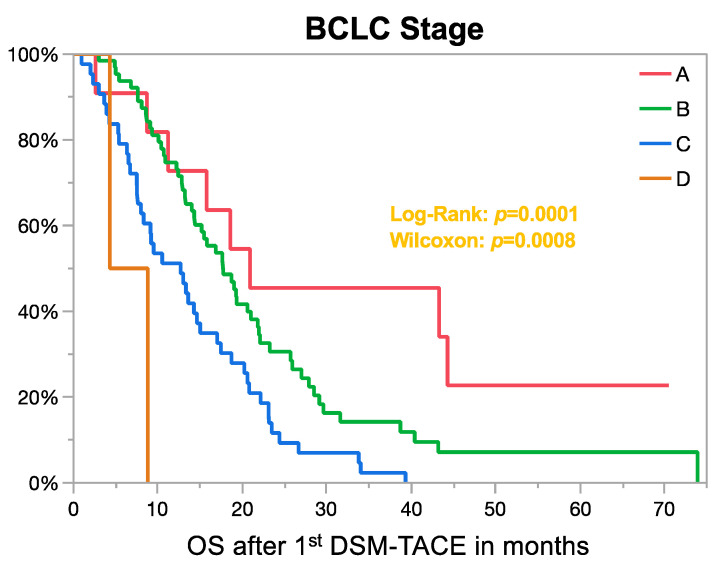
Overall survival (OS) according to the BCLC stage. OS was stratified by the BCLC stage following the first DSM-TACE. OS was statistically significant between patients with BCLC stages B and C (*p* = 0.003). Differences between A and B (*p* = 0.1) or between C and D (*p* = 0.1) were not statistically significant.

Median overall survival (OS) of all patients was 15.5 months (95% CI: 13.2–18.7 months) (Figure 1A. There was no statistical difference regarding the OS between institutions (Log-Rank: *p* = 0.06; Wilcoxon: *p* = 0.51) with 17.6 months (95% CI: 8.3–27) for Berlin, 16 months (95% CI: 12.7–20.8) for Essen, and 15.2 (95% CI: 10.9–18.6) for Rome (Figure 1B). OS according to the BCLC stage is graphed in Figure 2.

Univariate analysis identified several pretreatment characteristics to be associated with longer survival rates (Table 3). Here, patients with a lower Child–Pugh class (A/B/C: 17/15.2/8.95 months), lower BCLC stage (A/B/C/D: 20.9/17.7/12.7/6.6 months), unilobar disease (19 vs. 13.6 months for bilobar), absence of vascular invasion (16.9 vs. 13.8 months for vascular invasion) and absence of extrahepatic metastases (17.7 vs. 11.2 months with metastases) survived significantly longer. Patients with HCC lesions smaller than 10 cm (long axis) also survived significantly longer than patients with at least one HCC lesion larger than 10 cm (16.9 vs. 11.5 months). It may be noted that with cut-offs of 2, 5, and 7 cm, no statistical differences between groups could be observed. No overall correlation between the absolute size of the largest tumor size and the OS could be shown (r^2^: 0.01, *p* = 0.28). Similarly, the absolute number of HCC lesions was not a statistically significant factor affecting OS in the correlation analysis (r^2^: 0.018, *p* = 0.14).

Multivariate analysis with all significant UVA values was performed and could identify tumors smaller than 10 cm and unilobar disease as independent prognostic factors for more prolonged survival (Table 3). Survival was independent of the chemotherapeutic agent used (*p* = 0.34).

Neither the embolization pattern (whole liver, lobar, selective), chemotherapeutic drug used, nor adding Lipiodol (if any was given in at least in one session) were significant factors regarding OS (Table 4). Patients who received subsequent therapy (*n* = 50) after DSM-TACE survived significantly longer (18.7 months vs. 13.3) with a lower hazard ratio (HR: 0.6, 95% CI: 0.4–0.9; *p* = 0.01) in UVA.

### 3.4. Response Analysis

**Figure 3:**
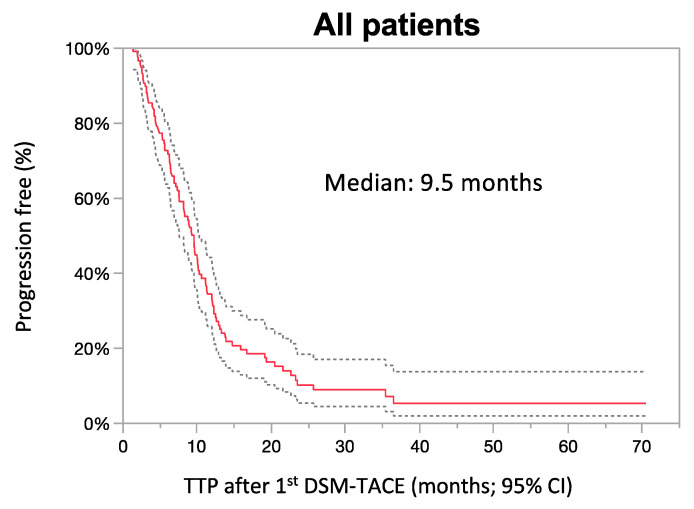
Time to progression (TTP) after the first treatment. TTP of all patients following the first DSM-TACE treatment incl. 95% confidence interval (95% CI).

Response analysis was available for 119 (98.3%) patients, as two died before the first response assessment imaging. The median TTP was 9.5 months (95% CI: 7.6–10.3) (Figure 3). The best achieved response was complete response in 13.5% (*n* = 16), partial response in 44.5% (*n* = 53), stable disease in 25.2% (*n* = 30), and progressive disease in 16.8% (*n* = 20). Best response was recorded after a median of 3 (range: 1–6) treatments with a median of 4 (1–6) for CR, 3 (1–6) for PR, 2.5 (1–4) for SD, and 2 (1–4) for PD (r^2^: 0.085, *p* = 0.0013). Nevertheless, it must be acknowledged that imaging was not routinely performed during the first three treatments, potentially biasing the analysis. Patients with a complete response had the longest TTP, with a median of 21.5 months, followed by a partial response (months 9.5), stable disease (9.7 months) and progressive disease (2.9 months), *p* < 0.0001. In total, six patients (5%) could subsequently undergo liver transplantation after achieving a complete response in four of the patients. One patient could undergo resection following successful downstaging.

### 3.5. Safety Analysis

Clinical adverse events (AEs) according to the CIRSE classification were recorded in 15.8% for Grade 1, 0.36% for Grade 2 and 0.9% for Grade 3. Grade 1 complications were abdominal pain (10%), nausea (3.6%), vomiting (0.9%) and post-embolization syndrome (1.25%). Grade 2 complications were nausea (0.2%), and burning (0.2%), and Grade 3 complications were duodenal ulcer (0.2%), cholecystitis (0.2%) and fatigue (0.5%).

Complications with permanent post-procedure sequelae or occurring deaths were not observed. Laboratory AEs according to the CTCAE v5 are recorded in Table 5, showing only limited numbers of grade III/IV AEs with up to 7.1% and 0.71% for grade III and IV AST increases, respectively. Overall, major laboratory AEs were lower or non-existent. It may be noted that assuming pretreatment anticoagulation affecting prothrombin time for all patients, only Grade 1 AEs for INR would have occurred.

**Figure 4:**
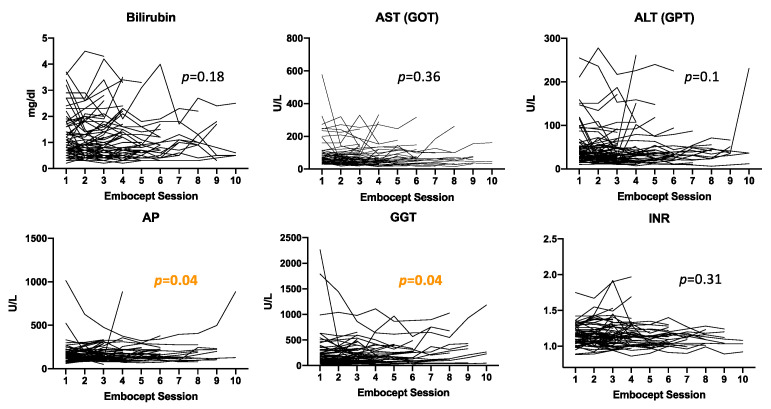
Laboratory changes over time. Laboratory values before each treatment session are graphed for individual patients. Laboratory values graphed are AST (aspartate-aminotransferase); ALT (alanine-aminotransferase); AP (alkaline phosphatase); GGT (gamma-glutamyltransferase); and INR (international normalized ratio).

Analysis of laboratory changes over time of pretreatment laboratory values, as shown in Figure 4, demonstrated that liver function remains stable over time, only showing significant alterations (increase or decrease) of GGT and AP in individual patients. However, no overall increase could be detected.

## 4. Discussion

Our study represents the largest European multicenter series on the use of chemoembolization with degradable starch microspheres (DSM-TACE) in a selective population with HCC. Our findings obtained from 558 treatments performed in 121 patients showed that DSM-TACE is a safe and effective treatment alternative in a “real-world” scenario when other palliative treatment alternatives fail or cannot be pursued due to assumed elevated risks. Moreover, the included patients were also at high risk of treatment failure or liver function decompensation, being characterized by a very high tumor burden (bilobar disease in 63.6% and >3 HCC nodules in 61.2%), with extrahepatic metastases in more than 20% of patients. Among the patients, 32 (26.4%) had vascular invasion with portal and/or hepatic vein thrombosis, 44 (36%) had ascites, 42 (34.7%) were Child–Pugh B/C class, 53 (43.8%) had a total bilirubin level higher than normal, 32 (26.5%) were more than 2 mg/dL and 15 (12.4%) were more than 3 mg/dL. The highest total serum bilirubin level observed was 5.1 mg/dL. Taking these advanced conditions in mind, with an overall objective response rate (CR, PR) of 57%, a median TTP of 9.5 months and a median OS of 15.5 months, with no significant side effects permanent postprocedural sequelae or occurring deaths, the outcomes are promising. Additionally, in six patients, HCC lesions could be downstaged with DSM-TACE to with subsequent liver transplantation. Similarly, Orlacchio et al. also demonstrated the feasibility of DSM-TACE for downstaging and bridging liver transplantation [7,17]. As research showed that patients are suitable for liver transplantation even when beyond Milan criteria, the number of patients suitable for transplantation may even increase in the future [18].

As expected, patients with advanced BCLC stage and a higher Child–Pugh class, a more extensive liver tumor manifestation (>10 cm, bilobar disease, portal vein invasion) as well as an extrahepatic manifestation experienced a shorter OS. However, only extensive tumors >10 cm and bilobar disease remained significant on multivariate analysis, lowering the other pretreatment factors’ role in OS. Considering that tumor size and lobar involvement are associated with tumor burden, our findings are in accordance with the findings from another study where tumor burden has been recognized as the most relevant prognostic factor for all palliative treatment options (intra-arterial therapy, sorafenib, best supportive care) [19].

When comparing the achieved survival of DSM-TACE to no treatment, the comparison suggests a survival benefit for DSM-TACE: the previously reported median OS of 600 Italian HCC patients treated with best supportive care was 9 months for all patients with 25 months for BCLC stage A, 10 months for stage B, 7 months for stage C and 6 months for stage D [20]. In comparison, median OS according to BCLC A/B/C/D were 20.9/17.7/12.7/6.6 months in our study, respectively. The placebo group (vs. Sorafenib treatment) in the SHARP and Asian Pacific trial mainly consisted of BCLC C patients (83–96.1%) with BCLC stage B of the other patients [21,22]. Here, the placebo groups had a median OS of 4.2 (BCLC C) and 7.9 months (BCLC B). In comparison, patients in our cohort with BCLC B (*n* = 8) and BCLC C (*n* = 11) who underwent a prior treatment attempt with sorafenib had a median OS of 19.3 and 9.2 months following DSM-TACE, respectively. Thus, in patients with BCLC B and C, data suggest a prolonged survival for DSM-TACE compared to best supportive care. Regarding Child–Pugh class, patients with Child–Pugh B receiving placebo/best supportive care instead of systemic treatment had a reported median OS within the range of 3.5–8.0 months, which was substantially lower than the achieved survival of 15.2 months when treated with DSM-TACE, thus suggesting a survival benefit [23,24,25].

DSM-TACE could also be compared to yttrium-90 transarterial radioembolization (SIRT) due to the similar patient clinical settings considered in published SARAH [26] and SIRveNIB [27] trials, both designed to show superiority comparing SIRT to sorafenib in advanced patients. An OS of 8.8 months was obtained in the SIRT group in both trials, substantially lower than our achieved survival. The cost-effective analysis could also be another point potentially favoring DSM-TACE when compared with SIRT. It would be interesting to underline that SIRT is generally contraindicated in patients with serum bilirubin levels > 2 mg/dL and/or decompensated cirrhosis (Child–Pugh ≥ B8). Based on these two formal criteria only, 43 patients (35.5%) of our study population would not be amendable to SIRT. These patients survived a median of 15.8 months (95% CI: 9.3–20.2), which is similar to the rest of our cohort (15.2 months, 95% CI: 12.8–19.3; *p* = 0.38). Thus, DSM-TACE also represents a promising treatment option for patients, even when SIRT is contraindicated.

The recently published “LiverT” study highlighted that a meaningful proportion of patients treated with a single TACE would experience substantial liver deterioration not only directly following the treatment but also in the long-term follow-up (30–90 days) [28]. After treatment with DSM, only a limited number of laboratory AEs were recorded, with few major AEs. Additionally, repetitive treatment can be performed safely with no tendency to overall liver deterioration. However, it must be acknowledged that findings may be subject to selection bias, as patients experiencing liver deterioration may have been allocated to a different treatment or palliative care.

In contrast to conventional and DEB-TACE and SIRT, DSM-TACE needs to be repetitively performed until the tumor cannot be controlled anymore or any other cause warranting treatment discontinuation. Before prematurely abandoning DSM-TACE as an effective treatment option, it must be considered that at least three (with up to six treatments) should be attempted.

The role of the added chemotherapeutic agent remains controversially discussed. Several studies concluded that, when treated with one chemotherapeutic agent, the treatment efficacy was comparable between agents, which was similar to our results [29,30]. On the other hand, a network meta-analysis suggests using a drug combination, including the combination of doxorubicin with mitomycin c. As only a few patients in this study cohort received this combination, further evaluation would be warranted if a combination treatment could further improve survival and response rates.

Despite the varying inclusion criteria among institutions, DSM-TACE was performed in patients in whom an alternative treatment, at the time of tumor board consensus, was not considered appropriate, and thus DSM-TACE was chosen as the treatment option. Moreover, by performing uni- and multivariate as well as subgroup analyses, the differences between the study groups were accounted for by identifying independent prognostic factors and thus promoting the understanding of the strengths and limitations of DSM-TACE as described and discussed above for various subgroups.

In summary, repetitive DSM-TACE is a veritable treatment option for all HCC patients with (I) high/diffuse tumor burden; (II) not suitable for or failing other curative or palliative treatment options; (III) serum bilirubin level of up to 3 mg/dL; and (IV) limited extrahepatic disease not prognostically relevant compared to liver involvement. As further drugs and treatment combinations such as Atezolizumab + Bevacizumab and a multitude of multikinase inhibitors become available, the role of DSM-TACE in the treatment algorithm warrants further investigation [31].

This study has several limitations. Due to its retrospective nature, the study underlies a risk of reporting bias, potentially limiting the findings of this study. Additionally, the additional use of Lipiodol to achieve the endpoint was not standardized and not commonly performed at all participating institutions, thus warranting further investigation. As patients from several institutions were included with varying inclusion and exclusion criteria, the current study cohort is more diverse without a clear overall cohort definition. On the other hand, this study with its mixed population may represent a more “real-world” patient cohort reflecting the clinical routine.

## 5. Conclusions

Transarterial chemoembolization with DSM is an effective alternative palliative treatment option for patients with a high tumor burden not suitable for or failing other therapies. Moreover, repetitive DSM-TACE preserves liver function over time, even in patients whose liver is treated as a whole.

## Figures and Tables

**Table 1:** Patient baseline characteristics.

Baseline Characteristic	Number of Patients (%)
Cirrhosis	109 (90%)
Etiology of cirrhosis	
Alcohol	33 (30.3%)
Viral	41 (37.6%)
Mixed	15 (13.8%)
Other	18 (16.5%)
Unknown	2 (1.8%)
Ascites	
None	77 (64%)
Mild	15 (12%)
Moderate to severe	29 (24%)
Encephalopathy	
None	119 (98.3%)
Grade I–II	2 (1.7%)
Disease extent	
Bilobar	77 (63.6%)
Unilobar	44 (36.4%)
Number of lesions	
Uninodular	17 (14%)
2–3 nodules	30 (24.8%)
Multinodular (>3 nodules)	74 (61.2%)
Largest lesion (standard deviation; range)	4 cm (±4.3; 0.8–24.7 cm)
Vascular invasion	32 (26.4%)
No	89 (73.6%)
PVTT (vp1–3)	28 (23.1%)
PVTT (vp1–3) + HVTT (vv3)	1 (0.83%)
PVTT (vp4)	1 (0.83%)
PVTT (vp4) + HVTT (vv2)	1 (0.83%)
HVTT (vv2)	1 (0.83%)
Limited extrahepatic metastases	27 (22.3%)
Child–Pugh-class	
A	79 (65.3%)
B	37 (30.6)
C	5 (4.1%)
BCLC stage	
A	11 (9.1%)
B	64 (53.9%)
C	43 (35.6%)
D	3 (2.5%)
ECOG	
0	55 (46%)
1	31 (25.6%)
2	7 (5.8%)
3	2 (1.7%)
Unknown	26 (21.5%)
Pretreatment	61 (50.4%)
Resection	19
Ablation	21
DEB-TACE ^a^	21
cTACE ^b^	3
Radioembolization ^c^	19
Sorafenib	20
Liver transplantation	1
Radiation therapy	1
Systemic chemotherapy	1

Patients may have received more than one prior therapy. ^a^ DEB-TACE was performed a median of two treatments per patient (range 1–3) with selective administration of 70–150 μm (27.1%), 100–300 μm (59.5%) or 300–500 μm (13.5%) beads. Moreover, 50 mg epirubicin (67.6%) or doxorubicin (32.4%) were used for DEB-TACE. ^b^ cTACE was performed once per patient in a selective manner with mitomycin c (0.85–2.75 mg) with 1.3–4.1 mL Lipiodol. ^c^ Radioembolization was performed in a bilobar (33%; median: 3 GBq, range: 1.9–4.92 GBq), lobar (25%; median: 3 GBq, range: 1.8–5 GBq) or segmental (42%; median: 1.4 GBq, range: 1.2–1.7 GBq) approach. Abbreviations: BCLC (Barcelona Clinic Liver Cancer); cTACE (conventional transarterial chemoembolization); DEB-TACE (drug-eluting bead transarterial chemoembolization); ECOG (Eastern Cooperative Oncology Group); GBq (gigabecquerel); PVTT (portal vein tumor thrombus); HVTT (hepatic vein tumor thrombus); SD (standard deviation).

**Table 2:** Laboratory values before the first DSM-TACE treatment.

Laboratory Value	Normal Range	ULN > 1 to 2	ULN > 2
INR (0.8–1.2)	62.8%	37.2%	-
Bilirubin (0.1–1.2 mg/dL)	56.2%	25%	18.8%
Creatinine (0.8–1.2 mg/dL)	78.1%	21%	0.9%
AST (5–40 IU/L)	32.7%	38.5%	28.9%
ALT (7–56 IU/L)	71.4%	18.5%	10%
AP (44–147 IU/L)	61.2%	32.8%	6.1%
GGT (8–38 IU/L)	5.1%	30.3%	64.7%
AFP (10–20 ng/mL)	45.6%	10.1%	44.3%

Laboratory values before the first DSM-TACE treatment with stratification regarding the normal range and times of the upper level of normal (ULN). Abbreviations: AFP (Alpha-Fetoprotein); ALT (alanine-aminotransferase); AP (alkaline phosphatase); AST (aspartate-aminotransferase); GGT (gamma-glutamyltransferase); INR (international normalized ratio).

**Table 3:** Uni- and multivariate overall survival analysis of pretreatment factors.

				Univariate Analysis		Multivariate Analysis	
Subgroups		Number of Patients	Median OS in Months (95% CI)	HR (95% CI)	*p*-Value	HR (95% CI)	*p*-Value
Gender	Female	23	20.8 (10.4–33.8)	0.62 (0.38–1.03)	0.06	-	-
	Male	98	14.4 (12.8–17.6)	1		-	
Ascites	No	77	17.4 (14.3–20.6)	0.7 (0.47–1.03)	0.07	-	-
	Yes	44	13 (8.7–19)	1		-	
Number of nodules	Uninodular	30	16.9 (9.1–27.9)	1.47 (0.8–2.7)	0.39	-	-
	2–3 nodules	17	13.3 (10.1–26.7)	1.2 (0.77–1.9)		-	
	Multinodular	74	17.4 (12.7–19)	1		-	
Largest liver lesion	≤5 cm	72	15.5 (12.8–19)	0.82 (0.55–1.22)	0.33	-	-
	>5 cm	49	14.3 (7.6–18.7)	1		-	
Largest liver lesion	≤7 cm	93	16.9 (13.3–20.3)	0.69 (0.44–1.08)	0.1		-
	>7 cm	28	12.7 (7.5–17.7)	1			
Largest liver lesion	≤10 cm	109	16.9 (13.3–19.3)	0.38 (0.2–0.7)	0.006	0.34 (0.17–0.68)	0.002
	>10 cm	12	11.5 (3–17)	1		1	
Lobar involvement	Unilobar	44	19 (14–22.2)	0.52 (0.33–0.78)	0.0022	0.63 (0.4–0.98)	0.042
	Bilobar	77	13.6 (10.9–17.4)	1		1	
Vascular invasion	No	89	16.9 (13.3–19.3)	0.57 (0.38–0.88)	0.013	0.96 (0.48–1.38)	0.9
	Yes	32	13.8 (7.6–18.7)	1		1	
Child–Pugh class ^a^	A	79	17 (13.2–20.6)	0.23 (0.08–0.67)	0.021	0.3 (0.07–1.34)	0.17
	B	37	15.2 (10.4–19)	0.34 (0.12–1.003)		0.42 (0.1–1.9)	
	C	5	8.95 (4.3–10.9)	1		1	
BCLC stage ^b^	A	11	20.9 (8.9–.)	0.1 (0.02–0.5)	0.0005	0.24 (0.03–2.1)	0.43
	B	64	17.7 (14.3–21.8)	0.2 (0.04–0.7)		0.3 (0.05–2.6)	
	C	43	12.7 (7.7–15.2)	0.3 (0.07–1.32)		0.48 (0.05–4.1)	
	D	3	6.6 (4.3–8.8)	1		1	
Prior therapy	No	60	14.6 (12.4–20.8)	1.3 (0.89–1.9)	0.19	-	-
	Yes	61	15.8 (11.2–19.3)	1		-	
Extrahepatic metastases	No	94	17.7 (14–20.2)	0.46 (0.29–0.73)	0.002	0.6 (0.35–1.03)	0.07
	Yes	27	11.2 (8–14.3)	1		1	

Results from Kaplan–Meier analysis and uni- and multivariate survival analysis of pretreatment factors are shown. ^a^ CP-Class: only the difference between A and C was statistically significant in UVA subgroup analysis (*p* = 0.007). ^b^ BCLC stage: In the subgroup analysis, A vs. B and C vs. D were not statistically significant. Abbreviations: 95% CI (95% confidence interval); BCLC (Barcelona Clinic Liver Cancer); HR (hazard ratio).

**Table 4:** Survival analysis of treatment properties.

				Univariate Analysis	
Subgroups		Number of Patients	Median OS in Months (95% CI)	HR (95% CI)	*p*-Value
Chemotherapeutic drug ^a^	Epirubicin	43	17.7 (13.3–21)	0.91 (0.62–1.4)	0.34
	Doxorubicin	75	13.6 (11.2–17.6)	1	
	Doxorubicin + Mitomycin C	3	19.3 (17.7–.)	0.43 (0.11–1.7)	
Embolization pattern ^a^	Selective	49	15.5 (11.2–19.25)	1	0.12
	Unilobar	39	17.6 (9.1–23.3)	0.7 (0.43–1.1)	
	Bilobar	33	14.3 (9.5–20.6)	1.12 (0.71–1.78)	
Lipiodol added ^b^	No	89	15.8 (13–18.7)	1	0.64
	Yes	32	14.2 (7.6–21)	1.1 (0.71–1.75)	

Uni- and multivariate survival analysis regarding treatment properties. ^a^ In the subgroup analyses, no differences between each subgroup were detected. ^b^ Lipiodol added was considered positive if Lipiodol was given in at least one treatment session.

**Table 5:** Laboratory adverse events.

Parameter	Grade	All Patients	Bilobar	Lobar	Selective	Pearson Correlation (*p*-Value)
**Bilirubin** **(*n* = 266)**	0	129 (48.5%)	47 (46.5%)	62 (55.4%)	20 (61%)	*p* = 0.28
	1	69 (25.9%)	32 (31.6%)	32 (28.6%)	5 (15.2%)	
	2	64 (32%)	19 (18.8%)	18 (16.1%)	7 (21.2%)	
	3	4 (1.5%)	3 (3%)	-	1 (3%)	
	4	-	-	-	-	
**AST** **(*n* = 282)**	0	131 (46.5%)	40 (40%)	69 (49.6%)	22 (50%)	*p* = 0.18
	1	98 (34.8%)	32 (32%)	54 (28.8%)	12 (27%)	
	2	31 (11%)	16 (16%)	9 (6.5%)	6 (13.6%)	
	3	20 (7.1%)	10 (10%)	6 (4.3%)	4 (9.1%)	
	4	2 (0.71%)	1 (1%)	1 (0.7%)	-	
**ALT** **(*n* = 242)**	0	159 (65.7%)	68 (68.7%)	77 (70%)	14 (42.4%)	*p* = 0.006
	1	70 (28.9%)	24 (24.2%)	29 (26.4%)	17 (51.2%)	
	2	7 (2.9%)	6 (6.1%)	0 (0%)	1 (3%)	
	3	6 (2.4%)	1 (1%)	4 (3.6%)	1 (3%)	
	4	-	-	-	-	
**GGT** **(*n* = 244)**	0	236 (96.7%)	93 (92.1%)	111 (100%)	32 (100%)	*p* = 0.003
	1	8 (3.3%)	8 (7.9%)	-	-	
	2	-	-	-	-	
	3	-	-	-	-	
	4	-	-	-	-	
**AP** **(*n* = 238)**	0	227 (95.4%)	97 (97%)	99 (93.4%)	31 (96.9%)	*p* = 0.43
	1	11 (4.6%)	3 (3%)	7 (6.6%)	1 (3.1%	
	2	-	-	-	-	
	3	-	-	-	-	
	4	-	-	-	-	
**INR** **(*n* = 240)**	0	230 (97%)	95 (96.9%)	103 (97.2%)	32 (97%)	*p* = 0.99
	1	7 (3%)	3 (3.1%)	3 (2.8%)	1 (3%)	
	2	-	-	-	-	
	3	-	-	-	-	

Laboratory adverse events according to the Common Terminology Criteria for Adverse Events (CTCAE) v5.0. Abbreviations: AST (aspartate-aminotransferase), ALT (alanine-aminotransferase), AP (alkaline phosphatase), GGT (gamma-glutamyltransferase), and INR (international normalized ratio).

## Data Availability

Data from this study can be found in supplementary material.

## Supplementary Materials

The following are available online at https://www.mdpi.com/article/10.3390/cancers13205122/s1, Study data.
